# Primary adrenal insufficiency: New genetic causes and their long‐term consequences

**DOI:** 10.1111/cen.14109

**Published:** 2019-10-30

**Authors:** Federica Buonocore, John C. Achermann

**Affiliations:** ^1^ Genetics & Genomic Medicine UCL Great Ormond Street Institute of Child Health University College London London UK

**Keywords:** Addison's disease, adrenal, adrenal insufficiency, genetics, revertant mosaicism, sphingolipidosis, steroidogenesis

## Abstract

Primary adrenal insufficiency (PAI) is a potentially life‐threatening condition that requires urgent diagnosis and treatment. Whilst the most common causes are congenital adrenal hyperplasia (CAH) in childhood and autoimmune adrenal insufficiency in adolescence and adulthood, more than 30 other physical and genetics cause of PAI have been reported. Reaching a specific diagnosis can have implications for management and for monitoring associated features, as well as for counselling families about recurrence risk in siblings and relatives. Here, we describe some recent insights into the genetics of adrenal insufficiency and associated molecular mechanisms. We discuss (a) the role of the nuclear receptors DAX‐1 (*NR0B1*) and steroidogenic factor‐1 (SF‐1, *NR5A1*) in human adrenal and reproductive dysfunction; (b) multisystem growth restriction syndromes due to gain‐of‐function in the growth repressors CDKN1C (IMAGE syndrome) and SAMD9 (MIRAGE syndrome), or loss of POLE1; (c) nonclassic forms of STAR and P450scc/*CYP11A1* insufficiency that present with a delayed‐onset adrenal phenotype and represent a surprisingly prevalent cause of undiagnosed PAI; and (d) a new sphingolipidosis causing PAI due to defects in sphingosine‐1‐phosphate lyase‐1 (*SGPL1*). Reaching a specific diagnosis can have life‐long implications for management. In some situations, milder or nonclassic forms of these conditions can first present in adulthood and may have been labelled, “Addison's disease.”

## 1 INTRODUCTION

Primary adrenal insufficiency (PAI) is a relatively rare but potentially life‐threatening condition that can result from a broad range of causes. In adulthood, the most common aetiologies include autoimmune “Addison's disease,” haemorrhage, infiltrative disorders/metastases and infection, whereas congenital adrenal hyperplasia (usually due to 21‐hydroxylase deficiency) affects approximately 1:18 000 infants and children.^1^, ^2^, ^3^ In recent years, however, an ever‐expanding list of genetic causes of PAI has been established (Table 1; see also International Classification of Pediatric Endocrine Diagnoses; http://www.icped.org). These conditions can have different inheritance patterns and potentially important associated features. Furthermore, milder or nonclassic forms of some of these conditions may only first present in teenage years or adulthood.

**Table 1:** Overview of causes of primary adrenal insufficiency with a focus on monogenic conditions

**Developmental disorders (Hypoplasia)**	**Congenital adrenal hyperplasia (CAH)**
aX‐linked adrenal hypoplasia (*NR0B1*/DAX‐1)	Congenital lipoid adrenal hyperplasia (*STAR*)
bSteroidogenic factor‐1 related (*NR5A1*/SF‐1)	P450scc (*CYP11A1*)
cIMAGe syndrome (*CDKN1C*)	3β‐hydroxysteroid dehydrogenase II (*HSD3B2*)
IMAGe‐like syndrome (*POLE1*)	21‐hydroxylase (*CYP21A2*)
dMIRAGE syndrome (*SAMD9*)	11β‐hydroxylase (*CYP11B1*)
SERKAL syndrome (*WNT4*)	17α‐hydroxylase/17,20‐lyase (*CYP17A1*)
Idiopathic (unknown)	P450 oxidoreductase (*POR*)
**Familial Glucocorticoid Deficiency (FGD) and related conditions**	**Metabolic causes**
MC2R (ACTH receptor) (FGD1)	Neonatal adrenoleukodystrophy (*PEX* genes)
MRAP (FGD2)	aX‐linked adrenoleukodystrophy (*ABCD1*)
Nonclassic STAR (“FGD3”)	Smith‐Lemli‐Opitz syndrome (*DHCR7*)
Nonclassic CYP11A1	Primary xanthomatosis (Wolman disease) (*LIPA*)
Minichromosome maintenance 4 (*MCM4*) Triple A syndrome (*AAAS*) Nicotinamide nucleotide transhydrogenase (*NNT*) Thioredoxin reductase (*TXNRD2*) bGlucocorticoid resistance (GR, N*R3C1*)	eMitochondrial disorders (esp. Mito DNA; *MRPS7*; *NDUFAF5*; *GFER*) Sphingosine‐1‐phosphate lyase‐1 (*SGPL1*)
**Disorders of aldosterone synthesis and action**	**Autoimmune adrenalitis (Addison disease)**
Aldosterone synthase (*CYP11B2*)	Autoimmune polyglandular syndrome type 1 (*AIRE*)
dMineralocorticoid resistance (MR, *NR3C2*)	Autoimmune polyglandular syndrome type 2
	Isolated autoimmune Addison disease
**Other nongenetic causes**	
Infections (TB, fungal, bacterial, HIV) Infiltrative (metastatic, amyloidosis, sarcoidosis, haemachromatosis) Drug effects Idiopathic (unknown)	

Note

Modified from the International Classification of Pediatric Endocrine Diagnoses (http://www.icped.org, Chapter 8A). All monogenic conditions listed are autosomal recessive unless indicated.

a X‐linked recessive.

b Autosomal dominant/de novo or autosomal recessive.

c Paternally imprinted (maternally expressed).

d Autosomal dominant/de novo.

e Mitochondrial DNA in some situations.

Here, we review some recent insights into the genetics and molecular mechanisms of rare forms of PAI and show how reaching a specific diagnosis can have implications for management and long‐term care. We will not discuss forms of CAH (including POR‐related syndromes), or well‐established adrenal insufficiency syndromes such as Triple A syndrome (achalasia, Addison's, alacrima) or X‐linked adrenoleukodystrophy.

## 2 NUCLEAR RECEPTORS IN ADRENAL DEVELOPMENT: DAX‐1 (NR0B1) AND SF‐1 (NR5A1)

DAX‐1 (officially termed NR0B1) and steroidogenic factor 1(SF‐1, officially termed NR5A1) belong to the nuclear receptor superfamily. Humans have 41 different nuclear receptors, 20 of which currently have associated clinical conditions.^4^ DAX‐1 and SF‐1 are the two nuclear receptors that regulate both adrenal and reproductive development and function.^5^

### 2.1 DAX‐1 (*NR0B1*)

Pathogenic variants in NR0B1/DAX‐1 were first reported as a cause of X‐linked adrenal hypoplasia congenita (AHC) in 1994.^6^ Efforts to localize the gene were helped by the co‐existence of adrenal hypoplasia with Duchenne muscular dystrophy due to a contiguous gene deletion syndrome on the short arm of the X chromosome (Xp21). More than 300 individuals and families with X‐linked AHC due to loss of NR0B1/DAX‐1 have been reported.^5^, ^7^

The classic clinical features of X‐linked AHC include primary salt‐losing adrenal insufficiency, hypogonadotropic hypogonadism (HH) and infertility. Boys may also present with predominantly either mineralocorticoid or glucocorticoid insufficiency, or may have paradoxical features such as macrophallia or early puberty.^8^, ^9^ One report of fertility in a man with X‐linked AHC using testicular sperm extraction‐intracytoplasmic sperm injection (TESE‐ICSI) has been published.^10^ Making the specific diagnosis is important so that associated features can be monitored and treated. The risk of presymptomatic adrenal insufficiency in brothers and males in the maternal family needs to be considered.^11^

**Figure 1:**
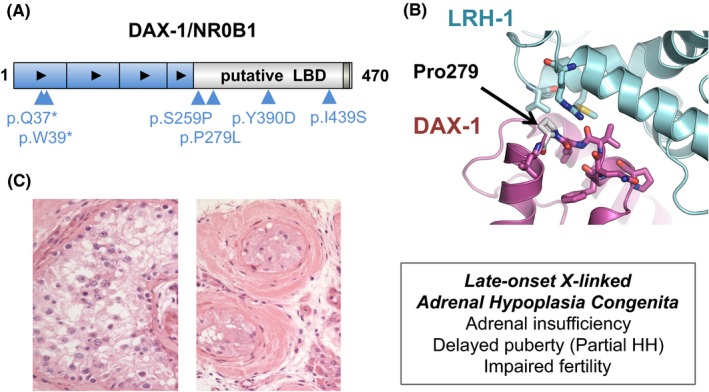
Late‐onset X‐linked adrenal hypoplasia congenita (AHC) due to variations in DAX‐1/NR0B1. (A) Cartoon showing the protein structure with selected variants associated with late‐onset adrenal insufficiency highlighted. Black arrowheads represent repeat motifs. (B) Model of DAX‐1 bound to LRH‐1, a homologue of steroidogenic factor‐1 (SF‐1/NR5A1), with Pro279 indicated. The amino acid change p.P279L alters a hydrogen bond at the periphery of a key interaction domain and is associated with a partial phenotype. Disruption of codon L278 at the core of the interaction domain is associated with classic early onset X‐linked AHC. (C) Selected testicular histology from men with late‐onset phenotypes includes oligospermia (*not shown*), maturational arrest at a primary spermatid stage (*left panel*); or more severe atrophic, hyalinized tubules, Sertoli cell only features and Leydig cell pseudohyperplasia (*right panel*). LBD, ligand binding domain; HH, hypogonadotropic hypogonadism. Panels (B) and (C) modified from Kyriakakis N, Shonibare T, Kyaw‐Tun J, et al Late‐onset X‐linked adrenal hypoplasia (DAX‐1, NR0B1): two new adult‐onset cases from a single centre. *Pituitary*. 2017;20(5):585‐593 ^©^ The Authors (http://creativecommons.org/licenses/by/4.0/)

Since 2000, several reports of late‐onset X‐linked AHC in men with PAI have emerged.^12^, ^13^, ^14^, ^15^, ^16^ Usually, this condition is associated with partial HH but infertility might be the main feature. Often the genetic change involves a 5' (aminoterminal) stop variant in the gene and translation of a shorter protein with partial function, or a partial loss‐of‐function variant in the ligand‐like binding domain of NR0B1 (Figure 1A, 1).^12^ One recent review of adult men with PAI in a single UK centre identified two patients with X‐linked AHC due to partial loss‐of‐function variants in DAX‐1, suggesting that late‐onset X‐linked AHC may be underdiagnosed in the adult population (Figure 1B & 1).^16^

### 2.2 SF‐1 (*NR5A1*)

Steroidogenic factor‐1 (NR5A1) (located on 9q33) is another nuclear receptor that regulates adrenal and reproductive development. Targeted deletion of the gene encoding Nr5a1 in the mouse causes adrenal and gonadal dysgenesis; children with a similar phenotype of adrenogonadal dysfunction were first reported in 1999 and 2002.^17^, ^18^, ^19^ These individuals had variants affecting key DNA‐binding regions of SF‐1 (P‐box and A‐box).^20^

Since these first publications, more than 250 individuals with pathogenic variants in NR5A1/SF‐1 have been reported.^5^ These changes are usually heterozygous de novo variants but can occur in a “sex‐limited dominant” pattern; in this situation, an unaffected woman carries a heterozygous change and passes it to affected 46,XY children, thereby resembling an X‐linked condition. Pathogenic SF‐1 variants are associated with a spectrum of phenotypes in 46,XY subjects including testicular dysgenesis/dysfunction (46,XY differences/disorders in sex development), severe hypospadias (accounting for approximately 5%‐7% of cases) and male factor infertility (1%‐2%).^21^, ^22^, ^23^, ^24^, ^25^, ^26^, ^27^, ^28^ Although data are limited, it has been proposed that this subset of infertile men could develop hypogonadism and low testosterone with time, so this may represent a group who need longer term endocrine follow‐up.^25^

Analysis of larger pedigrees where individuals with 46,XY testicular dysfunction were found together with 46,XX women with ovarian insufficiency (POI) has revealed that defects in SF‐1 can affect human ovary function too.^29^ Loss‐of‐function variants in NR5A1 are now well‐established in familial POI, but occur less commonly in sporadic (nonfamilial) POI or secondary amenorrhoea (1%‐2%).^22^, ^28^, ^29^, ^30^, ^31^, ^32^ Of note, variants in a specific amino acid in the A‐box of SF‐1 (p.R92) are found in 46,XX ovotesticular DSD (ovotestes or testis), suggesting that very localized alterations in this key transcriptional regulator can “switch” ovary development into a testis development pathway in humans.^33^, ^34^, ^35^

Despite these wide‐ranging effects on reproductive function, SF‐1 variants causing *adrenal dysfunction* are comparatively rare. Only six children have been reported to date to have SF‐1‐associated adrenal dysfunction, usually with variants in p.G35 or p.R92.^18^, ^19^, ^36^, ^37^, ^38^ It remains to be seen whether adrenal dysfunction will occur progressively in individuals with reproductive dysfunction due to defects in SF‐1; available insights currently suggest that this is not the case, although longer term systematic follow‐up studies are needed. Therefore, it seems that human gonadal function is more sensitive to haplo‐insufficiency or partial loss of SF‐1 activity than adrenal function.

## 3 COMPLEX MULTISYSTEM GROWTH DISORDERS: CDKN1C, SAMD9 AND POLE1

Adrenal insufficiency has also been reported as part of three recently described multisystem growth restriction disorders: IMAGe syndrome, MIRAGE syndrome and POLE1. Although relatively rare, these conditions are associated with interesting pathogenic mechanisms and may be underdiagnosed.

### 3.1 CDKN1C: IMAGe syndrome

IMAGe syndrome is characterized by intrauterine growth restriction, metaphyseal dysplasia, adrenal hypoplasia and genitourinary anomalies (often mild hypospadias) and was first described in 1999.^39^ The adrenal dysfunction can be variable, including both glucocorticoid and mineralocorticoid insufficiency, and diabetes mellitus has been reported in some members of a large kindred.^40^

**Figure 2:**
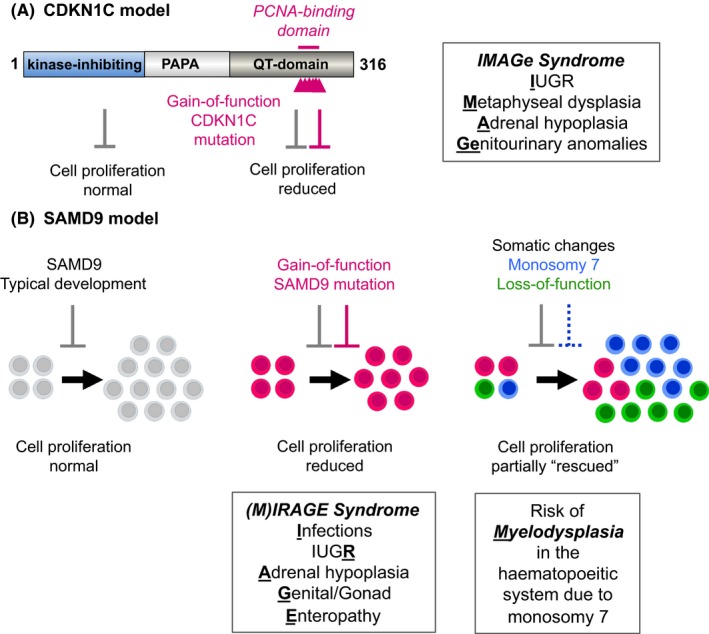
Complex multisystem growth disorders associated with gain‐of‐function mutations in CDKN1C and SAMD9. (A) CDKN1C is an inhibitor of cell cycle progression. Loss of CDKN1C is associated with the overgrowth condition, Beckwith‐Wiedemann syndrome, whereas gain‐of‐function mutations of CDKN1C cause adrenal hypoplasia as part of IMAGe syndrome. (B) SAMD9 also inhibits cell proliferation during normal foetal development. Gain‐of‐function mutations in SAMD9 (magenta) cause multisystem growth restriction as part of (M) IRAGE syndrome. Cells that develop somatic reversion events such as the monosomy 7 (blue) or loss‐of‐function mutations in SAMD9 (green) have a proliferative advantage and can partially “rescue” the phenotype. However, monosoomy 7 may be associated with secondary events, such as myelodysplasia in the haematopoietic system. IUGR, intrauterine growth restriction. Panel (B) modified from Buonocore F, Kühnen P, Suntharalingham JP, et al Somatic mutations and progressive monosomy modify SAMD9‐related phenotypes in humans. *J Clin Invest*. 2017;127(5):1700‐1713. ^©^ 2017 The Authors (http://creativecommons.org/licenses/by/4.0/)

IMAGe syndrome is usually caused by heterozygous missense variants in the key negative cell cycle regulator, cyclin‐dependent kinase inhibitor 1C (CDKN1C).^41^ These changes are localized to the PCNA‐binding domain and cause a gain‐of‐function and growth repression (Figure 2A).^41^, ^42^, ^43^, ^44^ The mechanism is unclear but may involve decreased degradation of CDKN1C, allowing prolonged cell cycle repression and delayed S‐phase progression. *CDKN1C* is an imprinted gene, which is only expressed from the maternal allele, so inheritance can mimic an X‐linked condition (although both boys and girls are affected). Interestingly, *loss‐of‐function* of this growth repressor, CDKN1C, is found in approximately 10% of patients with Beckwith‐Wiedemann syndrome (BWS), an “overgrowth” syndrome.^41^, ^45^ Children with BWS are at risk of adrenal tumours highlighting how developmental hypoplasia and cancer can sometimes be at opposite ends of a molecular spectrum.

### 3.2 SAMD9: MIRAGE syndrome

Another multisystem growth restriction disorder occurs due to gain‐of‐function variants in the growth repressor, Sterile Alpha Motif Domain Containing 9 (SAMD9) (Figure 2B).^46^, ^47^ Most children with this condition are born preterm with growth restriction and develop variable, salt‐losing adrenal insufficiency in early life, although a small proportion of children do not exhibit adrenal features. Penoscrotal hypospadias or more female‐typical genitalia can be seen in 46,XY children and other features occur, such as infections, enteropathy, respiratory distress, anaemia and thrombocytopaenia, and hydrocephalus. The condition has been termed MIRAGE syndrome (myelodysplasia [see below], infections, restricted growth, adrenal hypoplasia, gonadal anomalies and enteropathy).^46^ Mortality can be high.

Most gain‐of‐function SAMD9 variants occur de novo, although some germline inheritance and variable penetrance has been described.^46^, ^48^ SAMD9 may be involved in recycling growth factor receptors (eg EGFR) through endosome trafficking, so that gain‐of‐function reduces availability of these receptors and leads to growth restriction.

*SAMD9* is located on the long arm of chromosome 7 (7q21). One fascinating feature of this condition is that children with SAMD9 mutations who survive early infancy often develop monosomy 7, partial 7q deletions or somatic nonsense (stop gain) changes in SAMD9 in haematopoietic cell lines, which “remove” the mutant SAMD9 allele and confer a clonal growth advantage on those cells (Figure 2B).^46^, ^47^, ^49^, ^50^, ^51^ This phenomenon can rescue the blood phenotype in the short term. However, loss of 7q21 (including *SAMD9* and *SAMD9L*) can result in myelodysplastic syndrome in the bone marrow (the “M” in MIRAGE) and further somatic “hits” can sometimes lead to the development of leukaemia.^52^ In contrast, some children have different forms of revertant mosaicism, such as gene conversion or uniparental disomy, which replace the mutant SAMD9 allele with a wild‐type allele.^51^, ^53^ In these situations, the bone marrow features reverse and no haematopoietic issues develop.

It is hypothesized that such dynamic changes may modify the phenotype in different organs, explaining why some children have a mild or even no adrenal features.^47^ Indeed, somatic modulation and revertant mosaicism could play a wider role in the phenotypic of endocrine disorders than is currently recognized.

### 3.3 POLE1

Recently, biallelic loss‐of‐function variants in polymerase epsilon‐1 (POLE1, Pol ε) have been reported to cause an IMAGe‐like syndrome, in children with growth restriction and adrenal hypoplasia (with variable salt‐loss), together with variable immune dysfunction and distinctive facial features.^54^ A small proportion of children have preserved adrenal function. POLE1 is a key DNA leading‐strand polymerase, and most subjects reported to date have a heterozygous intronic variant (c.1686 + 32C>G) together with a disruptive loss‐of‐function variant in the other allele. POLE1 plays an important role in DNA replication by binding to PCNA and extending DNA synthesis in the replisome. Loss of POLE1 disrupts this mechanism and is associated with delayed S‐phase progression and cell division, although the exact mechanism is unclear.

## 4 NONCLASSIC STEROIDOGENIC DISORDERS: STAR AND CYP11A1

Steroidogenic acute regulatory protein (STAR) and cytochrome P450 side‐chain (P450scc, encoded by *CYP11A1*) are two key factors involved in the initial stages of steroidogenesis in the adrenal gland and gonads.^55^

**Figure 3:**
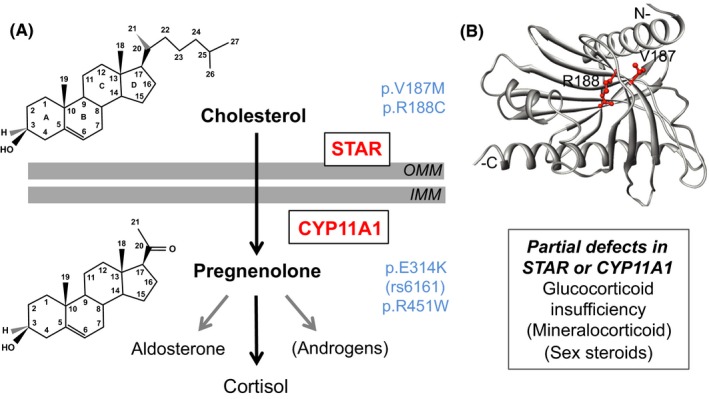
Partial defects in STAR and CYP11A1 cause a predominant glucocorticoid insufficiency phenotype. (A) Cartoon showing the actions of STAR and P450scc/CYP11A1 in steroidogenesis in the adrenal gland (and gonad). Selected variants associated with partial phenotypes are indicated. (B) Model of STAR indicating the position of codons V187 and R188 at the core of the protein, which interact with cholesterol. OMM, outer mitochondrial membrane; IMM, inner mitochondrial membrane. Panel (B) modified with permission from Baker BY, Lin L, Kim CJ, et al Nonclassic congenital lipoid adrenal hyperplasia: A new disorder of the steroidogenic acute regulatory protein with very late presentation and normal male genitalia. *J Clin Endocrinol Metab*. 2006;91(12):4781‐4785. ^©^ 2006 The Endocrine Society

STAR is located on the outer mitochondrial membrane and facilitates transfer of cholesterol from the cytoplasm to the mitochondrial inner membrane (Figure 3A). P450scc is the limiting step in steroidogenesis and catalyses the three steps needed for conversion of cholesterol to pregnenolone (Figure 3A).^55^

Severe disruption of STAR or P450scc/*CYP11A1* causes a block in all aspects of adrenal and gonadal steroid synthesis.^56^, ^57^, ^58^ For STAR defects this is known as congenital lipoid adrenal hyperplasia (CLAH).^56^ Individuals with these conditions tend to present in the first month of life with salt‐losing adrenal insufficiency and cortisol insufficiency. Infants with a 46,XY karyotype have female‐typical genitalia due to a lack of testosterone biosynthesis *in utero*. Ovarian insufficiency can occur variably in 46,XX girls at adolescence.

In addition to these “classic” conditions, it has now become apparent that partial defects in these enzymes cause nonclassic disorders; patients present with a predominant adrenal phenotype, usually affecting glucocorticoid synthesis and often resembling familial glucocorticoid deficiency (FGD).

### 4.1 Nonclassic congenital lipoid adrenal hyperplasia – STAR

Nonclassic congenital lipoid adrenal hyperplasia (NCLAH) was first reported in 2006 due to biallelic pathogenic missense variants in STAR.^59^ Two brothers had predominant glucocorticoid insufficiency and normal genitalia (p.R188C), and a 46,XX girl had low cortisol and extremely high ACTH (p.V187M) (Figure 3A). These variants reduce interactions with the 3‐βOH group of cholesterol and cause partial loss of function (Figure 3B). In some individuals, variants in other codons (eg p.R192, p.G221) are found.^37^, ^60^, ^61^ Given the overlap with features of familial glucocorticoid deficiency (resistance), NCLAH is sometimes termed FGD3. Long‐term reproductive follow‐up is required to ensure that puberty development occurs appropriately and adequate testosterone and oestrogen biosynthesis is maintained in adulthood. If semen analysis shows viable sperm, appropriate counselling and sperm banking might be offered in case progressive issues occur in the future.

### 4.2 Nonclassic CYP11A1 deficiency

Similar to NCLAH, it is now emerging that partial loss of function of *CYP11A1* (encoding P450scc) also presents with a predominant adrenal phenotype, affecting glucocorticoids and sometimes mineralocorticoid synthesis.^62^, ^63^ Patients can present at different ages throughout childhood, often with hyperpigmentation, hypoglycaemia or prolonged illness with infections.

Genetic analysis has revealed that many European individuals and families of European ancestry with this condition are compound (double) heterozygous for a c.940G > A variant (rs6161) on one allele of *CYP11A1* and a severely disruptive change on the other allele (Figure 3A).^64^ Although the c.940G>A variant is predicted to cause a benign protein change (p.E314K), detailed molecular studies have shown that it generates a novel splice site so that missplicing occurs.^64^, ^65^ This variant is carried by approximately 1:140 people of European descent, but is likely to cause adrenal insufficiency only when inherited with a very rare disruptive change on the other allele. Partial CYP11A1 insufficiency can also occur in other populations, such as due to the p.R451W variant common in central Turkey.^37^, ^63^

As with NCLAH, long‐term monitoring of sex hormone production and fertility is warranted at puberty and in adulthood. Fertility has been reported in men, although raised gonadotrophins are sometimes seen, and testicular adrenal rest tumours (TART) can occur if glucocorticoid insufficiency is poorly controlled.^66^ Sperm banking might also be considered.

## 5 A NEW SPHINGOLIPIDOSIS: SGPL1

Another recent discovery is the association of PAI with steroid‐resistant nephrotic syndrome, due to homozygous or compound heterozygous variants in sphingosine‐1‐phosphate lyase‐1 (SGPL1).^67^, ^68^, ^69^

**Figure 4:**
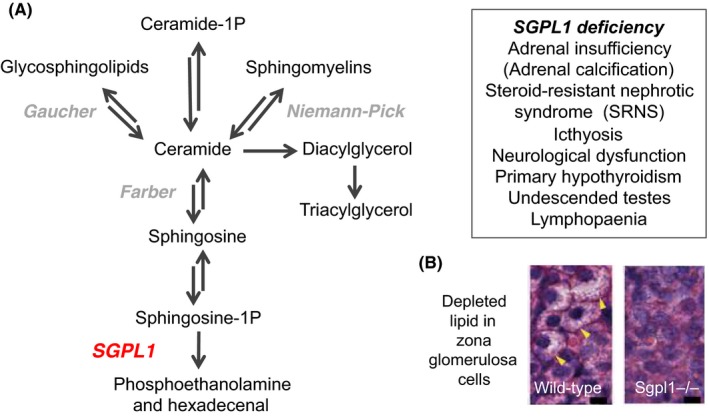
Sphingosine‐1‐phosphate lyase‐1 (SGPL1) disruption causes adrenal insufficiency. (A) SGPL1 regulates breakdown of ceramide, a pathway associated with several established metabolic disorders (sphingolipidoses). (B) The *Sgpl1^‐/‐^* knockout mouse has an adrenal phenotype including depleted lipid in zona glomerulosa cells. Panels (A) and (B) modified from Prasad R, Hadjidemetriou I, Maharaj A, et al Sphingosine‐1‐phosphate lyase mutations cause primary adrenal insufficiency and steroid‐resistant nephrotic syndrome. *J Clin Invest*. 2017;127(3):942‐953 ^©^ 2017 The Authors (http://creativecommons.org/licenses/by/4.0/)

SGPL1 is an enzyme that catalyses the breakdown of sphingolipids by cleaving sphingosine‐1‐phosphate.^67^ Disruption of this enzyme can lead to an accumulation of sphingolipids and ceramide and represents a novel sphingolipidosis (similar to Fabry disease, Gaucher disease and Niemann‐Pick disease; Figure 4A). Other clinical features reported in these patients include ichthyosis, neurological dysfunction, dyslipidaemia, lymphopaenia and other endocrine features such as primary hypothyroidism and cryptorchidism.^67^ The adrenal features are not invariable at presentation and may be masked by steroid treatment for nephrotic syndrome or precipitated by steroid withdrawal. Some children have mineralocorticoid insufficiency and *Sgpl1* knockout mice have depleted lipid in zona glomerulosa cells (Figure 4B). In patients, adrenal calcifications can sometimes be seen on imaging.^69^

SGPL1‐deficiency is an important diagnosis to make as it is a potentially progressive, multisystem disorder. Future therapies could target this pathway to restore function. Of note, SGPL1 antagonists have been trialled for the treatment of multiple sclerosis; initial data suggest these do not affect adrenal function but more information about the role of this pathway in the adrenal gland is needed.^70^

### 5.1 Genetic testing for PAI

Reaching a genetic diagnosis of PAI in childhood can have important implications for counselling and management, especially as inheritance patterns are variable, important potential associated features might need monitoring and treatment strategies can differ. Detecting affected family members before the onset of features can be important.

When presented with a child or young person with newly diagnosed adrenal insufficiency, several aspects of the history, clinical features or focused tests may give a clue to the underlying cause. For example:

1. Some more common causes of PAI such as congenital adrenal hyperplasia (CAH), autoimmune Addison's disease and some metabolic causes (eg X‐linked adrenal leukodystrophy) can be diagnosed by focused biochemical testing backed up (where relevant) by single gene testing. Features such as hyperandrogenism (CAH) or other autoimmune conditions may help;
2. Family history may point to an X‐linked condition (eg NR0B1/hypoplasia; ABCD1/adrenoleukodystrophy) if boys are affected in the maternal family, or a recessive condition if there is known consanguinity (Table 1);
3. Known associated features can be highly informative if present at diagnosis (eg hypogonadotropic hypogonadism and NR0B1/hypoplasia, steroid‐resistant nephrotic syndrome and SGPL1; IUGR/FGR and IMAGe/MIRAGE);
4. Ancestral background may be important as founder effects and geographical hotspots for some conditions are known.

In the absence of the potential features listed above, many forms of PAI have a similar biochemical profile (ie, elevated ACTH, low cortisol or poor cortisol response to stimulation, with or without mineralocorticoid insufficiency). Additional “clues” such as age of presentation and presence of salt‐loss can sometimes help with a likely broad diagnosis, but are not specific on their own. Therefore, next‐generation sequencing approaches using targeted panels of known genes or exomes are being increasing used in both research and clinical practice.

The diagnostic rate for known genes using some of these approaches is surprisingly high. In a national multicentre study of 95 children with PAI of unknown aetiology (non‐CAH, nonautoimmune, nonmetabolic) in Turkey, a genetic diagnosis was made in almost 90% of individuals, with variants in just 10 genes (eg MC2R, NR0B1 (DAX‐1), STAR, CYP11A1, MRAP, NNT, ABCD1, NR5A1, AAAS, SGPL1).^37^ Key founder effects and geographical hot spots were seen, such as a recurrent MRAP slice variant in Western Turkey and partial loss‐of‐function variant in CYP11A1 in Central regions. Obviously, high consanguinity rates would have enriched for recessively inherited conditions in this cohort, but even in an analysis of our data for young people presenting with PAI in the United Kingdom, a genetic diagnosis was reached in more than 60% of children and young people (unpublished data). Thus, genetic analysis seems worthwhile.

So, what is the best approach to clinical genetic analysis? This is a difficult question as access to genetic testing varies in different countries and policies change rapidly as technologies improve and costs reduce. In England, the “National Genomic Test Directory” was launched in March 2019 to provide guidance on commissioned genetic testing for rare and inherited diseases (https://www.england.nhs.uk/publication/national-genomic-test-directories/). At the time of writing, we would suggest that single gene testing is still preferred for conditions such as 21‐hydroxylase deficiency or X‐linked adrenoleukodystrophy where there are diagnostic biochemical markers. Focused panels are also available that include many of the genetic causes of PAI. Ultimately, in the future, clinical exomes or genomes with *targeted analysis* of relevant genes will likely be the best approach, as all known genes can be reviewed initially and, if the cause is not found, data can subsequently be reanalysed as new genetic causes are identified or the relevance becomes established of intronic changes that may affect splicing. Finally, knowledge of geographical hotspots can be very important for targeting genetic testing quickly and cost‐effectively, especially in resource‐limited settings.

As well as the known causes described here, gene discovery approaches using genome wide analysis, better understanding of human adrenal development and function, and newer genetic approaches should help in the discovery of additional causes of PAI in the future.^71^ Where no genetic cause is identified, other physical causes may have been overlooked. Finally, in a small but important group, adrenal insufficiency *resolves* and no specific cause is found.

## 6 CONCLUSIONS

The genetics of many rare forms of primary adrenal insufficiency is gradually being elucidated. Making a specific diagnosis can have implications for immediate management and for monitoring long‐term care. Milder or nonclassic forms of adrenal dysfunction can sometimes first present in teenage years or adulthood. In some situations, these individuals may have been labelled as having “Addison's” disease and more detailed genetic investigations to find a specific cause have not been undertaken.

## CONFLICT OF INTEREST

Nothing to declare.

## Data Availability

All data in this review are derived from published sources and are acknowledged or referenced accordingly.
